# The effect of fluorides in the TiO_2_(B) anode on the hydrogen evolution reaction in aqueous electrolytes

**DOI:** 10.3389/fchem.2026.1744630

**Published:** 2026-01-23

**Authors:** Khoi-Nguyen Nguyen, Lam Hoang Nguyen, Jozel John Salvacion, Nam Huu Nhat Nguyen, Samuel Ming Tuk Yeung, Seung Woo Lee, Liat Rosenfeld, Chengyu Song, Dahyun Oh

**Affiliations:** 1 Chemical and Materials Engineering Department, Charles W. Davidson College of Engineering, San José State University, San Jose, CA, United States; 2 George W. Woodruff School of Mechanical Engineering, Georgia Institute of Technology, Atlanta, GA, United States; 3 National Center for Electron Microscopy, The Molecular Foundry, Lawrence Berkeley National Laboratory, Berkeley, CA, United States

**Keywords:** aqueous lithium-ion batteries, fluorides, hydrogen evolution reaction, TiO2 nanorods, water-in-salt electrolytes, silanization

## Abstract

For aqueous lithium-ion batteries (A-LIBs), the hydrogen evolution reaction (HER) poses a significant challenge, as it competes with the primary electrochemical processes of the anode, resulting in capacity loss and reduced cycling stability. In this study, we investigate the use of fluorine-based additives in anodes to mitigate HER in LIBs with aqueous electrolytes including low or high amounts of salt (water-in-salt electrolytes (WiSE)). We synthesized and incorporated three distinct materials into TiO_2_(B) anodes: aluminum fluoride (AlF_3_), lithium fluoride (LiF), and 1H,1H,2H, 2H-perfluorooctyltriethoxysilane (FAS) using a solution-based method. Among these fluorides containing composite anodes, FAS containing anodes delayed HER onset potentials of WiSE by 45–160 mV (1.2 m (molality) or 21 m (Lithium bis (trifluoromethanesulfonyl) imide in H_2_O)) compared to the bare TiO_2_ (B) anodes. Among these fluorides, FAS demonstrated the highest HER delay with the smallest amount of additives due to its hydrophobic nature. These findings underscore the effect of fluorine-based passivation layers in mitigating the HER, potentially expanding the energy density, and improving the operational stability of anodes in A-LIBs, thereby paving the way for their broader application in sustainable energy storage.

## 1 Introduction

Aqueous lithium-ion batteries (A-LIBs) have emerged as a promising alternative to conventional LIBs that contain highly flammable organic electrolytes. In particular, the electrochemical stability window of water has been expanded from 1.23 V to 3 V with recent advancements using super-concentrated electrolytes such as water-in-salt electrolytes (WiSE) (Dong et al., 2025; Suo et al., 2015; Xie et al., 2020). WiSEs have a high concentration of lithium salts with anions containing fluorinated sulfone groups, enabling energy densities of A-LIBs to exceed 100 Wh/kg (Chen et al., 2020; Wang et al., 2016). Their enhanced performance is attributed to a small fraction of free water molecules in the electrolyte and the formation of a solid-electrolyte interphase (SEI) layer, protecting the anode from unwanted reactions (Yamada et al., 2016). Although the exact formation mechanism and composition of SEI layers remain unclear, LiF has been commonly observed in the SEI formed with WiSE, in addition to Li_2_O, Li_2_CO_3_, and CF_x_ (Dubouis et al., 2018; Suo et al., 2017). Despite these contributing factors for the enhanced stability windows, WiSE still suffers from their high cathodic limits, which restrict the use of low-voltage anodes and degrade cycling stability.

To reduce water electrolysis at the cathodic limit, previous efforts have been made to artificially construct SEI layers that can limit the access of water molecules while allowing Li-ion diffusion into the anode. Key parameters to consider in designing such artificial SEI layers include high Li-ion conductivity, low catalytic activity towards HER, and conformality. Based on these selection criteria, LiTi_2_(PO_4_)_3_, Al_2_O_3_, ZnO, or carbon were introduced as artificial SEI layers on anode via wet synthesis or atomic layer deposition (ALD) (Hou et al., 2021; Subramanya et al., 2020; Wang et al., 2020a). Another approach was to introduce LiF coatings, given their frequent occurrence in electrochemically developed SEI layers with WiSE. However, the LiF-based artificial SEI formed by ALD was not as effective as SEIs generated in contact with electrolytes in suppressing HER (Droguet et al., 2021).

Because the dominant transport mechanism in WiSE is based on Li^+^(H_2_O)_n_ motion proven by calculations, it is crucial to prevent the formation of hydrogen bonds between water and the electrode surface to minimize HER (Borodin et al., 2017). Fluorine is a poor hydrogen bond acceptor due to its higher electronegativity and lower polarizability compared to oxygen (Howard et al., 1996). Therefore, we hypothesize that surface coating with fluorides will create a less favorable environment for the formation of hydrogen bonds with water, thereby preventing HER. In this work, we utilized TiO_2_(B) nanorods (TNR), chosen for its distinct morphology and electrochemical activity, as a model system to evaluate the effectiveness of fluorides in delaying HER in aqueous electrolytes. Although TiO_2_(B) offers high energy density (Dylla et al., 2013), surface passivation is essential to mitigate its catalytic effect on hydrolysis. To test our hypothesis on the role of fluorides, we investigated three fluorides in TNR anodes: AlF_3_, LiF, and 1H,1H,2H,2H-perfluorooctyltriethoxysilane (FAS). AlF_3_ was chosen due to its higher ionic conductivity (AlF_3_ (amorphous): 8.8 × 10^−9^ S/cm at 25 °C) (Hao and Wolverton, 2013) but approximately five times lower solubility in water than LiF. LiF and FAS were selected for their respective advantages: LiF commonly appears in the SEI formed in WiSE, while FAS can easily interact with TNR due to the selective nature of silane bonding onto the hydroxyl groups of the metal oxide surface. Three fluoride-containing TNR composites were formed and their HER were evaluated in diluted (1.2 m of Lithium bis (trifluoromethanesulfonyl) imide (LiTFSI) in H_2_O) and concentrated (21 m of LiTFSI in H_2_O) aqueous electrolytes.

## 2 Materials and methods

### 2.1 Synthesis of TiO_2_(B) nanorods (TNR)

TNR were synthesized using a hydrothermal method (Yoshida et al., 2005). First, 1.25 g of TiO_2_ nanoparticles (99.9% anatase, 18 nm, United States Research Nanomaterials Inc.) were dispersed in 11.5 mL of 10 M NaOH (98%, Alfa Aesar) solution and vortexed for 5 min. The mixture was transferred into a 23-mL Teflon-lined autoclave and heated at 170 °C for 48 h. The resulting sodium titanate product was washed with deionized (DI, EMS, 18 MΩ cm) water twice and centrifuged at 9,000 rpm to lower the pH to 12. The product was then acid-treated by mixing with 30 mL of 0.1 M HCl (37%, Sigma-Aldrich), stirring for 24 h, and adjusting the pH to 2. After a second acid wash, the nanorods were neutralized to pH 7 by repeatedly rinsing with DI water using centrifugation. The nanorods were then dried in a vacuum oven at 70 °C overnight. The final product was calcined in air at 350 °C for 4 h with a heating rate of 5 °C/min, yielding TNR.

### 2.2 Synthesis of composites with TNR

First, aluminum nitrate nonahydrate (Al(NO_3_)_3_ · 9H_2_O, 99.997%, Sigma-Aldrich) or lithium acetate (C_2_H_3_LiO_2_, 99.95%, Sigma-Aldrich) solutions were prepared by dissolving 1 mmol of each in 10 mL of DI water. Also, the fluoride precursor solution was prepared by adding 1 mmol of ammonium fluoride (NH_4_F, >95%, Sigma-Aldrich) into 10 mL DI water. The Al (240 μL for 0.23 (mole fraction) AlF_3_-coated TNR) or Li (100 μL for 0.25 LiF-coated TNR) precursor solutions with the TNR (152 mg for AlF_3_-TNR and 180 mg for LiF-TNR, respectively) were stirred at 500 rpm for 120 h. After incubating the precursor solution with TNR for 5 days, 720 μL of NH_4_F precursor solution was then introduced by a syringe pump at 1 mL/min to the previously described mixture while stirring, and then heated to 80 °C on the hot plate. The solution was then heated to 100 °C until the solvent was evaporated, followed by drying the product at 70 °C under a vacuum. The dried powder is then annealed at 400 °C for 5 h in N_2_ (99.999%) to remove byproducts such as NH_4_NO_3_ (Julien and Mauger, 2019).

For the silanization of the TNR, 90 mL of ethanol (200-proof), 4 mL of 25% ammonia solution (EMSURE grade), and 0.15 mL of 1H,1H,2H, 2H-perfluorooctyltriethoxysilane (FAS) (98%, Sigma-Aldrich) were combined in a 100-mL Teflon liner and stirred at 500 rpm for 30 min. Simultaneously, 300 mg of TNR was sonicated in 10 mL of ethanol for 30 min. The FAS amount was chosen to yield molar ratios of approximately 10:1 (TNR:FAS) and 5:1 with respect to the total moles of TNR, allowing comparison of functionalization levels at different silane concentrations. The solutions were mixed together and placed in a silicone oil bath (85 °C–90 °C) with a chiller at 15 °C connected to the flask. The reaction proceeded for 3.5 h at 500 rpm. After completion, the FAS-TNR product was separated via centrifugation and washed once with a 1:1 (v/v) mixture of water and ethanol. The FAS-TNR product was dried in a vacuum oven at 70 °C overnight.

### 2.3 Material characterization

The phase of TNR was determined by X-ray Diffraction (XRD, Rigaku Ultima III X-Ray Diffractometer). Transmission Electron Microscope (TEM, 200 kV FEI mono-chromated F20 UT Tecnai at Lawrence Berkeley National Laboratory (LBL)) and Scanning Electron Microscope (SEM, Zeiss XB 550 FIB-SEM at the University of California, Berkeley) were used to examine the surface of the pristine and composite TNR samples. X-ray photoelectron spectroscopy (XPS, Thermo K-alpha, EAG Laboratories) was used with the monochromated Al K (alpha) X-ray source.

### 2.4 Electrochemical analysis

The electrolyte (1.2 m (molality) or 21 m of LiTFSI (98%, TCI) in DI water) was prepared by first drying the LiTFSI salt in a glovebox on a hot plate at 80 °C overnight. After drying, it was mixed with DI water on a hot plate (40 °C) for 1 hour. The Linear Sweeping Voltammetry (LSV) of TNR electrodes (working electrode, WE, 1 cm × 1 cm, weight ratio is 8:1:1 = TNR: PTFE: Carbon black) was conducted with a potentiostat (Biologic SP-150e) at a scan rate of 1 mV/s from −0.2 V to −1.8 V in an electrochemical cell including Ag/AgCl (4 M KCl) as the reference electrode (Pine research), and a graphite rod (3.5 cm in length with 0.5 cm in diameter) as the counter electrode. A second-order discrete differentiation method was used to determine the HER onset potential (de Falco et al., 2021). The Savitzky-Golay smoothing with a second-order polynomial fit was applied for the voltage windows that showed HER with the points of the window of ten, as larger windows (e.g., 20) tended to produce more positive onset potentials. The HER was defined as the voltage corresponding to the first largest negative (minimum) value of the second derivative. We measured the ohmic drop (R) between the reference and working electrode with electrochemical impedance spectroscopy before LSV test (EIS, from 1 Hz to 1 MHz, with 10 mV AC voltage), and the voltage was corrected as E_iR-corrected_ = E_measured_ - iR.

## 3 Results

**FIGURE 1:**
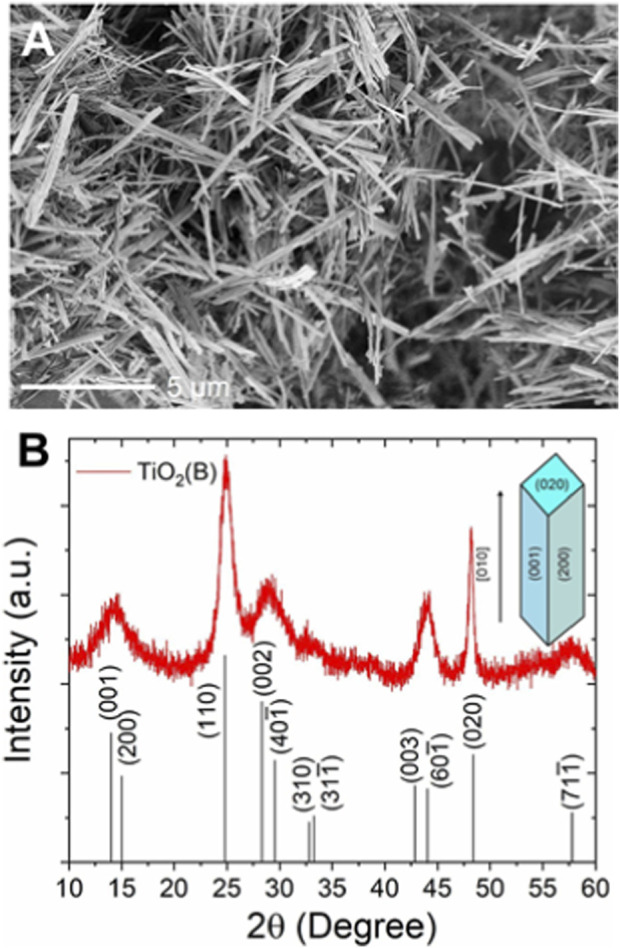
**(A)** SEM image and **(B)** XRD pattern (red) of TiO_2_ (B) nanorods (bottom: a reference XRD pattern of TiO_2_ (B) (JCPDS: 04-007-6246), inset: a schematic of the TNR with crystallographic planes labeled).

In this study, TNRs with diameters of approximately 100–200 nm and lengths of several micrometers were synthesized via a hydrothermal method (Figure 1A). The XRD results confirmed the successful synthesis of TiO_2_(B) by the characteristic peak at (001) (Figure 1B). The XRD peaks of TiO_2_(B) are broad due to the nanocrystalline nature of the nanorods. The sharpest peak at (020) suggests that TNR has a preferential crystal growth in (020), along the [010] direction (Figure 1B inset). In this work, TNR composites are named as AlF_3_-TNR, LiF-TNR, and S-TNR (TNR with FAS), respectively. The compositions of each composite are presented in Table 1, including their respective weight and mole fractions. We selected the mole fractions of AlF_3_ (0.23) or LiF (0.25) to have similar fractions across the composites. For S-TNR, compositions of 0.09 (10:1 = TNR:FAS, molar ratio) and 0.16 (5:1 = TNR:FAS, molar ratio) FAS were chosen to maintain a balanced level of electrode hydrophobicity for electrochemical measurements with aqueous electrolytes.

**TABLE 1:** Weight and mole fractions used for synthesis of LiF, AlF_3_, and silane (10:1 and 5:1) in composites. The weight fraction represents the ratio of the mass of each component to the total mass of the system, while the mole fraction indicates the ratio of the number of moles of each component to the total number of moles.

Component (A)	Weight fraction (W_A_/(W_A_ + W_TNR_))	Mole fraction (m_A_/(m_A_ + m_TNR_))
AlF_3_	0.24	0.23
LiF	0.1	0.25
Silane	0.00064	0.09
	0.00128	0.16

**FIGURE 2:**
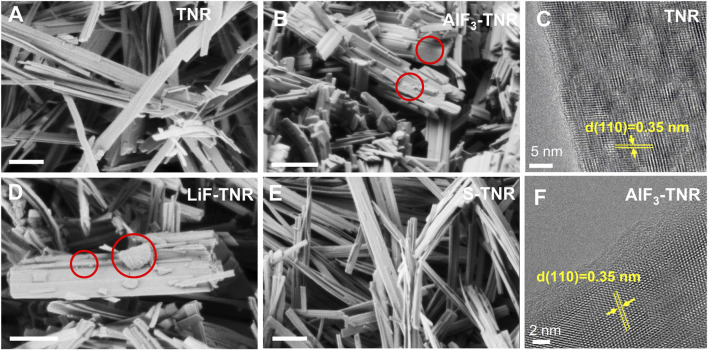
SEM images of **(A)** bare TNR, **(B)** AlF_3_-TNR, **(D)** LiF-TNR, and **(E)** S-TNR. LiF-TNR and AlF_3_-TNR composites showed particle-like structures (marked with red circles) loaded on the surface of TNR compared to the smooth surface of bare TNR or S-TNR, indicating the growth of inorganics (Scale bar: 500 nm). TEM images of **(C)** bare TNR and **(F)** AlF_3_-TNR.

We confirmed the formation of AlF_3_ (Figure 2B; Supplementary Figure S1B) and LiF (Figure 2C; Supplementary Figure S1C) particles on TNR with SEM images (Figure 2; Supplementary Figure S1) followed by their chemical analysis using XPS (more precise and larger sample area selection than SEM Energy Dispersive X-ray Spectroscopy (EDX)) that will be discussed in the following paragraph. It is evident that numerous AlF_3_ and LiF particles have been deposited on the surface of TNR, suggesting a notable growth of the inorganics compared to bare TNR (Figure 2A; Supplementary Figure S1A). In particular, we observed larger particles (up to 300 nm) in LiF-TNR composites, whereas AlF_3_-TNR composites showed smaller particles scattered on the TNR.TNR was incubated with Al or Li precursors for 5 days, and NH_4_F was introduced slowly using a syringe pump to promote the fluoride growth on the surface of TNR. In contrast, the surface of S-TNR exhibited a similar structure to that of the bare TNR (Supplementary Figure S1D) and their SEM images showed no particles or aggregates at the low magnification. We also observed serious charging with S-TNR during SEM imaging, indicating a homogeneous coating of the organic material on TNR. This observation highlights the distinct characteristics contributed by the inorganic and organic components. For AlF_3_-TNR, the TiO_2_(B) phase was still preserved although it involves an annealing process at 400 °C for 5 h, as confirmed by TEM image showing the d-spacings of 0.35 nm corresponding to (110) plane of TiO_2_(B) (Figures 2C,F).

**FIGURE 3:**
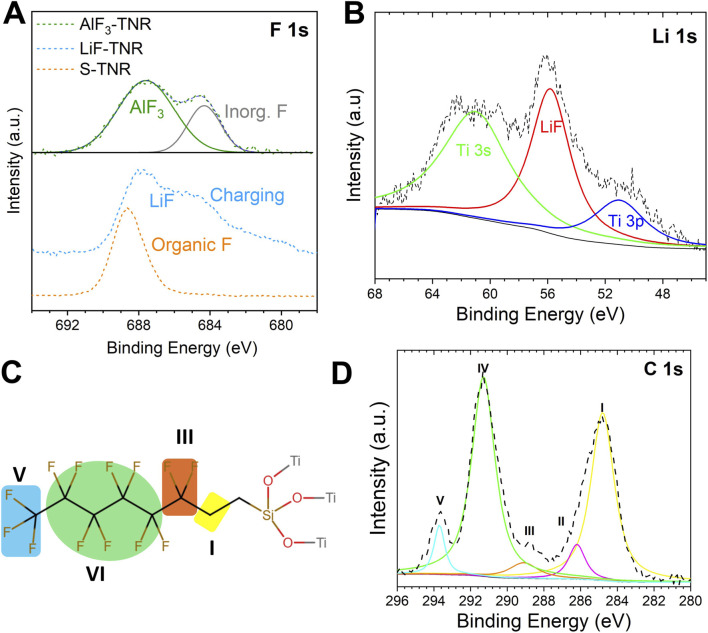
High energy resolution XPS spectra of composite TNR: **(A)** F 1s of AlF_3_-TNR, LiF-TNR, and S-TNR, **(B)** Li 1s of LiF-TNR, **(C)** A color-coded indication of the different chemical groups in FAS after grafting on TNR, and **(D)** C 1s of S-TNR.

The surface composition and chemical state of TNR composites were studied with XPS, confirming the successful synthesis of AlF_3_-TNR, LiF-TNR, and S-TNR. The F1s spectra from these composites confirmed the presence of AlF_3_, LiF, and FAS in the composites. First, the AlF_3_-TNR composite exhibited two distinct F 1s peaks originating from AlF_3_ (Hess et al., 1994) and inorganic fluorides (684.3 eV, Figure 3A) which may be attributed to unreacted fluoride precursors, as their positions are similar to the peak appearing in LiF-TNR composite. Although the LiF-TNR composite exhibited significant sample charging (broadened the peak and introduced shoulders) potentially due to inhomogeneous particle distribution, its F 1s peak is positioned at the binding energy of LiF (684.8 (Murch and Thorn, 1980) -686.5 (Ro and Linton, 1992) eV, Figure 3A). Its Li 1s spectrum (Figure 3B) also includes a distinct peak (55.7 eV) centered at the position of LiF, although it has a spectral overlap with the Ti 3p signal (Morgan et al., 1973). The S-TNR composite exhibited a distinct F1s peak at 688.6 eV (Figure 3A), in agreement with a prior report on FAS-modified surfaces (Yoo et al., 2018).

A closer inspection of the high-resolution C 1s spectrum revealed multiple distinct peaks of FAS at binding energies of 284.8, 286.2, 289.1, 291.3, and 293.8 eV (Figure 3D), supporting a successful grafting of FAS on TNR. The peak at 284.8 eV corresponds to adventitious carbon and aliphatic -CH_2_- groups commonly found in silane molecules, while the peak at 286.2 eV is indicative of oxidized carbon, C-O-, due to moisture exposure (Wan et al., 2011; Zhou et al., 2022). The peak at 289.1 eV is attributed to fluorinated carbon species, particularly the CH_2_-CF_2_ group, which is consistent with the molecular structure of FAS (Wang et al., 2020b). Notably, the strong peaks at 291.3 and 293.8 eV are characteristic of -CF_2_ and -CF_3_ groups, respectively, confirming the presence of perfluorinated chains from the FAS molecule on the TiO_2_ surface (Hozumi et al., 1999). The -CF_2_ and -CF_3_ groups are expected to enhance the hydrophobicity of the TNR surface. Further evidence for successful surface functionalization was observed in the Si 2p peak centered at 102.2 eV (Supplementary Figure S2), which is typically assigned to siloxane-type Si-O-Ti bonding environments, confirming the formation of covalent bonds between the silane group and the TiO_2_ surface (Parrino et al., 2025).

**FIGURE 4:**
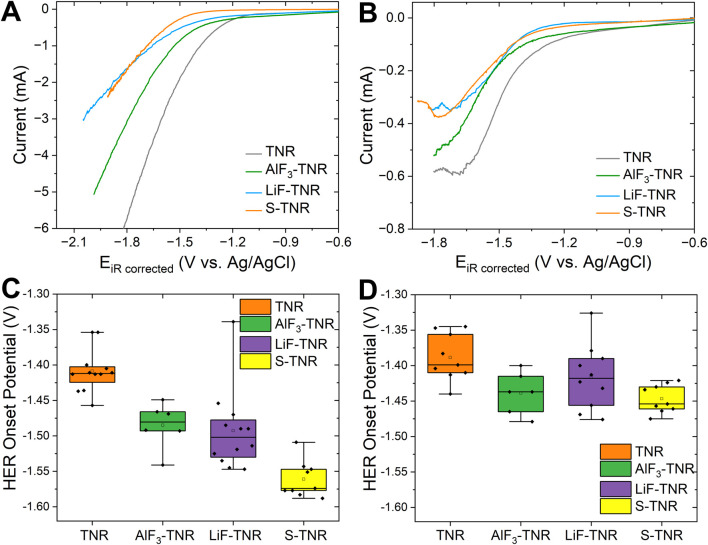
LSV scans of bare and composite TNR electrodes (AlF_3_-TNR, LiF-TNR, and S-TNR (10:1)) in **(A)** 1.2 m and **(B)** 21 m of LiTFSI/H_2_O electrolytes (reference electrode: Ag/AgCl, counter electrode: graphite rod, scan rate: 1 mV/s). **(C,D)** The HER onset potentials (C: 1.2 m and D 21 m) corresponding to the LSV scans (The number of replicates ranges from 4 to 9).

The cathodic limits of aqueous electrolytes (1.2 m and 21 m LiTFSI/H_2_O) with bare or composite TNR electrodes were measured using linear sweeping voltammetry (LSV) (Figure 4, all potential values in this manuscript have been iR-corrected and are reported relative to the Ag/AgCl reference electrode). The selection of these two electrolyte concentrations aimed to elucidate the relative effectiveness of fluorides in suppressing HER, particularly due to differences in the amount of free water. These differences are evident in the current responses observed in the LSV scans for the 1.2 m and 21 m LiTFSI/H_2_O electrolytes, showing a higher HER current from 1.2 m than 21 m (Figures 4A,B). A noticeable result is that the bare TNR electrode exhibited a high HER onset potential (representative iR-corrected potential values near the mean: 1.413 V (vs. Ag/AgCl) for 1.2 m and −1.399 V for 21 m) compared to the composite TNR electrodes in both electrolytes. This highlights the effectiveness of fluorides in delaying the HER onset potential.

A second-order discrete differentiation method was employed to determine the HER onset potentials for both pristine and coated/composited TNR electrodes, aiming to minimize subjective bias in the selection process. The data distribution among replicates for each electrode type is presented as box plots in Figure 4C (1.2 m) and 4D (21 m). LiF–TNR showed the largest variance in HER onset potentials, which is potentially due to the larger particles observed after LiF–TNR composite formation. Notably, all composites appear effective in delaying the HER onset potential by approximately 80–160 mV for 1.2 m but a small extent (15–45 mV) for 21 m from the bare TNR electrode (AlF_3_-TNR: (−1.492 V (1.2 m) and −1.437 V (21 m) LiF-TNR: −1.49 V (1.2 m) and −1.423 V (21 m) and S-TNR: −1.574 V (1.2 m) and −1.454 V (21 m)). This result also highlights the role of additives in delaying HER at low concentrations (1.2 m), which show more negative shifts than at 21 m.

## 4 Discussion

Between inorganic fluoride composites, the AlF_3_-TNR and LiF-TNR, they both presented similar HER onset potential, indicating the less significant effect of their solubility difference in HER onset potential. Among three composites, the S-TNR electrode was most effective in delaying HER in both 1.2 and 21 m electrolytes compared to two inorganic fluoride composites. In particular, we observed that S-TNR electrodes were generally more resistant to wetting within the electrolyte, unlike the AlF_3_-TNR or LiF-TNR electrodes (Supplementary Figure S4). Considering that less than 0.1 wt% of FAS in the S-TNR electrode is sufficient to delay HER, it presents an ideal choice for surface protection against HER compared to the inorganic composites. For all electrodes, we did not observe any LiTFSI reduction reaction that occurs around −0.577 V (Ag/AgCl) as a small plateau, especially with the low-concentration electrolytes (Suo et al., 2015).

**FIGURE 5:**
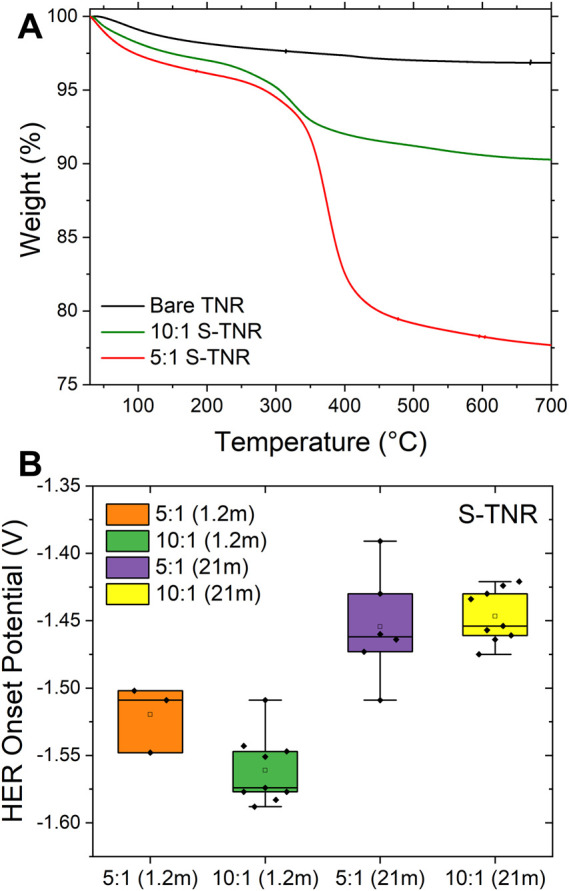
**(A)** TGA curves of bare and S-TNR with varied silane amounts. **(B)** The HER onset potentials of 5:1 and 10:1 S-TNR tested with 1.2 m and 21 m LiTFSI/H_2_O electrolytes (The number of replicates ranges from 3 to 9).

S-TNR electrodes exhibited a significantly lower current response at high potentials (from −0.6 V to the HER onset) with the 1.2 m electrolyte, demonstrating their effectiveness in limiting water access to the electrode. This performance contrasts with the AlF_3_-TNR and LiF-TNR electrodes, which have only partial coverage on the TNR surface. This is based on the tendency of water to avoid disrupting its hydrogen-bonded network on surfaces that lack hydrophilic sites (Dalvi and Rossky, 2010). Consequently, an ordered interfacial water layer forms, tightly bound to the fluorinated surface. This ordered water layer not only reduces the interaction between bulk water molecules from the aqueous electrolyte and the TiO_2_ anode, but also maintains a confined hydrogen-bond network, enhancing the surface’s macroscopic hydrophobicity (Zhang et al., 2021). We increased the amount of FAS (from 10:1 to 5:1 (TNR:FSA)) to evaluate its effectiveness on the HER onset potential. The amount of FAS was determined by thermogravimetric analysis (TGA, Figure 5A), showing 19.2% weight loss from 200 °C to 700 °C mainly due to FAS. The minimal weight loss below 200 °C is primarily attributed to the evaporation of physically adsorbed water on the TiO_2_ surface, consistent with previous thermal studies on TiO_2_ nanomaterial (Zhu et al., 2012). The untreated TNR exhibited only a small additional weight loss of 1.3%, resulting in a total weight loss of 3.1% by 700 °C. This minor loss is primarily due to the condensation of hydroxyl (-OH) groups at elevated temperatures, which is characteristic of metal oxide nanomaterials.

Increasing the concentration of silane (5:1 = FAS:TNR) results in similar HER suppression to 10:1 in the 21 m electrolyte, but results in a 65 mV performance decrease in the 1.2 m electrolyte system (Figure 5B). This trend is likely due to the detrimental impact of excessive silane concentration on the quality of the coating. At elevated concentrations, silane molecules are more prone to undergo self-condensation reactions (Si-O-Si) in the bulk solution, rather than silane-coupling to the TNR surface. This may result in the formation of thicker, non-uniform films rather than smooth coatings, thereby reducing the effectiveness of surface passivation. (Fadeev and McCarthy, 2000; Tian et al., 2021). Therefore, the superior HER suppression observed at the 10:1 ratio may be attributed to its ability to form a more uniform and adherent FAS layer. In contrast, the 5:1 formulation likely underperforms due to the formation of overly thick, poorly adhered coatings, a problem that is further exacerbated in the 1.2 m electrolyte system.

This study demonstrates the successful application of fluoride- and fluorine-based coatings of LiF, AlF_3_, and FAS onto TiO_2_(B) nanorods, improving the anode stability in A-LIBs. These coatings effectively mitigate hydrolysis and parasitic side reactions by suppressing the hydrogen evolution reaction on the anode surface, a marked improvement compared to pristine TiO_2_(B) electrodes. The introduction of fluorine-containing coatings imparted hydrophobicity to the anode surface, with FAS demonstrating superior protection against HER due to its preferred interaction with metal oxides surface compared to the fluoride-based composites. Our findings confirm that these surface modifications not only reduce hydrolytic interactions but also preserve the structural integrity of TiO_2_(B), resulting in improved charge-discharge cycling stability of TNR in aqueous electrolytes. Overall, this work underscores the potential of fluoride- and fluorine-based passivation layers to enhance the energy density and operational stability of anodes in A-LIBs, paving the way for their broader adoption in sustainable energy storage applications. Future research should focus on advanced synthesis techniques and exploring alternative passivation layers to further optimize the anode-electrolyte interface, ultimately contributing to the development of future aqueous battery technologies.

## Data Availability

The raw data supporting the conclusions of this article will be made available by the authors, without undue reservation.
