# Supplementary figures and images for: The effect of fluorides in the TiO2(B) anode on the hydrogen evolution reaction in aqueous electrolytes
Source: Front Chem. 2026 Jan 23;14:1744630. doi: 10.3389/fchem.2026.1744630 (PMC12877796; doi:10.3389/fchem.2026.1744630)

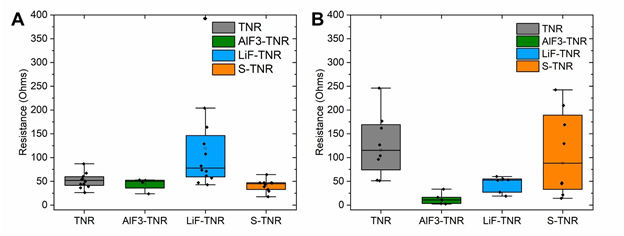

Supplement: Supplementary file 1 [file Image3.tif]

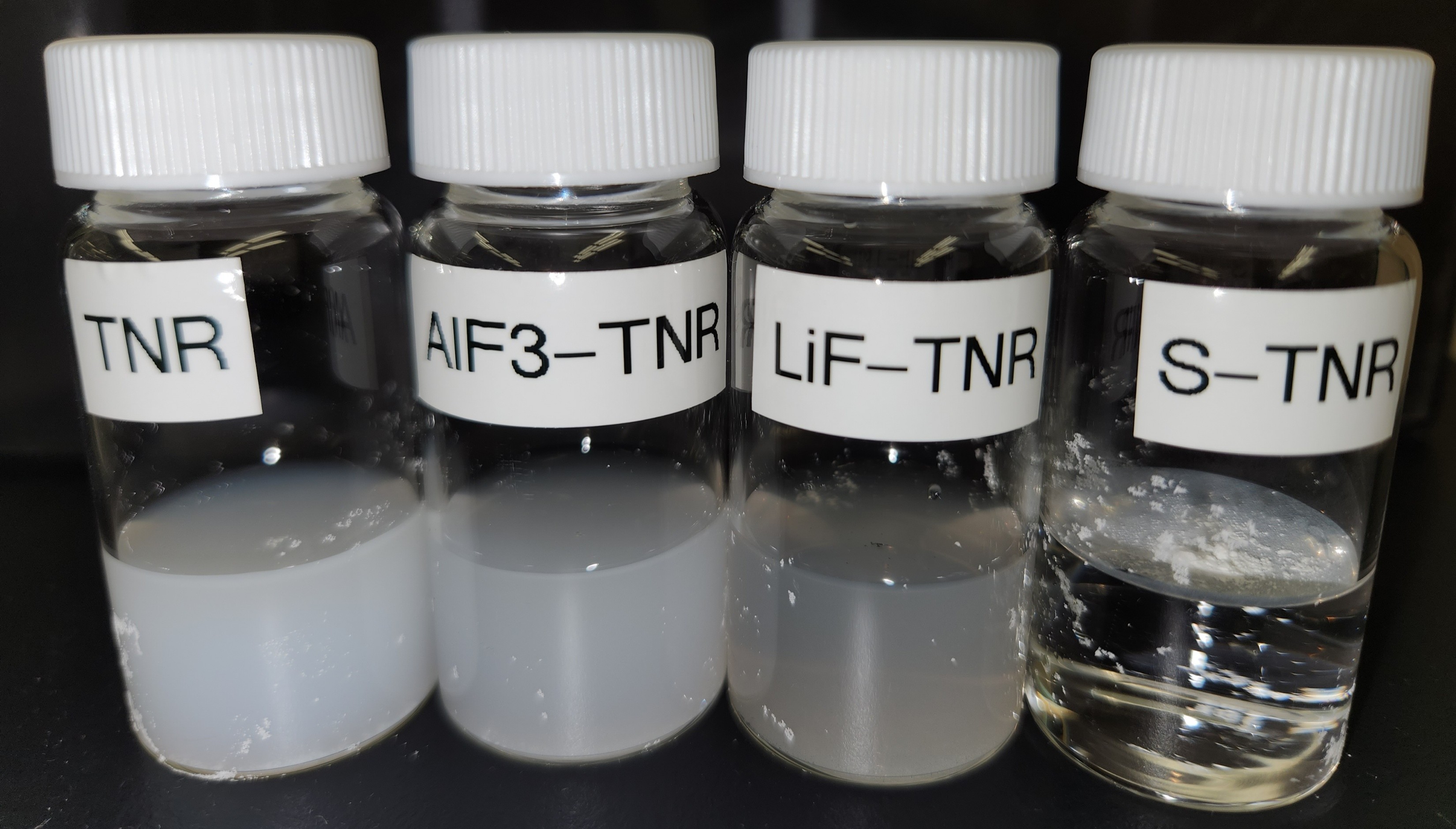

Supplement: Supplementary file 2 [file Image4.jpeg]

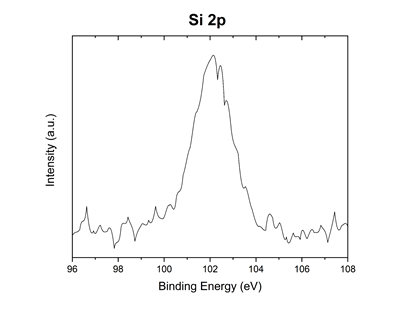

Supplement: Supplementary file 3 [file Image2.tif]

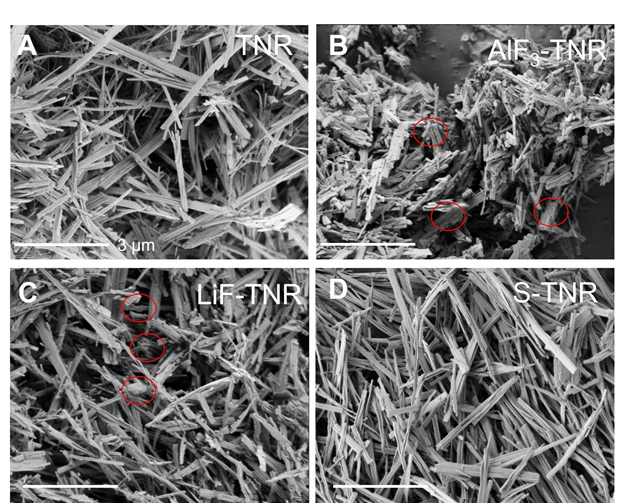

Supplement: Supplementary file 4 [file Image1.tif]
