# Supplementary material for: The effect of fluorides in the TiO2(B) anode on the hydrogen evolution reaction in aqueous electrolytes
Source: Front Chem. 2026 Jan 23;14:1744630. doi: 10.3389/fchem.2026.1744630 (PMC12877796; doi:10.3389/fchem.2026.1744630)
Supplement: Supplementary file 5 [file DataSheet1.pdf]

Supplementary Material

1 Supplementary Figures and Tables

1.1 Supplementary Figures

Supplementary Figure 1. SEM images showing the microstructural morphology at low magnification for (A) pure TNR, (B) AlF_3-TNR composite, (C) LiF-TNR composite, and (D) S-TNR composite. Red circles highlight the particles depositing on the TNR surface, specifically (B) LiF particles and (C) AlF_3 particles. Each image includes a scale bar of 3 μm for reference.

Supplementary Figure 2. High energy resolution XPS spectra of Si 2p S-TNR composite.

Supplementary Figure 3. Resistance plot of bare and coated TNR in A) 1.2m and B) 21m WiSE.

Supplementary Figure 4. Composites in deionized water showing different solubility.
